# Erythromycin resistance in Streptococcus pyogenes in Italy.

**DOI:** 10.3201/eid0602.000212

**Published:** 2000

**Authors:** M Bassetti, G Manno, A Collidà, A Ferrando, G Gatti, E Ugolotti, M Cruciani, D Bassetti

**Affiliations:** Infectious Diseases Department, University of Genoa, G. Gaslini Children's Hospital, Genoa, Italy. mattba@tin.it

## Abstract

In a prospective study of acute pharyngitis in Italian children, 69 (38.3%) of 180 isolates of Streptococcus pyogenes were resistant to macrolides. S. pyogenes was eradicated in 12 (63.1%) of 19 patients with erythromycin-resistant S. pyogenes treated with clarithromycin and in 22 (88%) of 25 patients with erythromycin-susceptible strains. The constitutive-resistant phenotype was correlated with failure of macrolide treatment.


### Erythromycin Resistance in *Streptococcus pyogenes* in Italy

**Matteo Bassetti,* Graziana Manno,* Andrea Collidà,* Alberto Ferrando,* Giorgio Gatti,* Elisabetta Ugolotti,* Mario Cruciani,† and Dante Bassetti***

*University of Genoa, G. Gaslini Children's Hospital, Genoa, Italy; and †Department of Infectious Diseases, Verona, Italy

In a prospective study of acute pharyngitis in Italian children, 69 (38.3%) of 180 isolates of *Streptococcus pyogenes* were resistant to macrolides. *S. pyogenes* was eradicated in 12 (63.1%) of 19 patients with erythromycin-resistant *S. pyogenes* treated with clarithromycin and in 22 (88%) of 25 patients with erythromycin-susceptible strains. The constitutive-resistant phenotype was correlated with failure of macrolide treatment.

Over the last decade, severe infections due to *Streptococcus pyogenes* and its complications have reemerged in several parts of the world (1-3). *S. pyogenes* is uniformly susceptible to penicillin, which remains the drug of choice for treating infections by this organism. Erythromycin and other macrolides have been recommended as alternative treatment for patients allergic to penicillin (2,4); however, resistance to erythromycin and related drugs in *S. pyogenes* has become widespread (5). Resistance to erythromycin was first described in 1955 in the United Kingdom (6) and, more recently, has been reported in Japan (7), Finland (8), Taiwan (9), Australia (10), the United States (11), Spain (12,13), and Italy (14-16).

From 1991 to 1996 in Genoa, the percentage of *S. pyogenes* resistant or with intermediate resistance to erythromycin increased from 0% to 50% (17). This abrupt increase in the rate of erythromycin-resistant strains is of concern, since erythromycin has been effective against most *S. pyogenes* isolates.

We investigated the prevalence and distribution of macrolide resistance phenotypes among *S. pyogenes* and carried out a clinical study in patients with *S. pyogenes* pharyngitis to correlate clinical and microbiologic outcomes with in vitro susceptibility patterns.

Address for correspondence: Matteo Bassetti, Infectious Diseases Department, University of Genoa, G. Gaslini Children's Hospital, Largo G. Gaslini 5, 16147 Genoa, Italy; fax: 39-010-392-614; e-mail: mattba@tin.it.

### The Study

Ten pediatricians in Genoa (population 700,000) participated in this study. Children included in the study had to have two or more of the following signs and symptoms: oropharyngeal erythema, fever and sore throat, tonsillar exudate or cervical lymphadenitis, and strawberry tongue.

*S. pyogenes* was confirmed by culture of throat swabs in agar blood; ß-hemolytic colonies were identified as *S. pyogenes* by the bacitracin disk (Difco Laboratories, Detroit, MI) and latex-agglutination test (Streptex, Wellcome, U.K.). Minimum inhibitory concentrations (MICs) for penicillin, cefixime, ceftriaxone, chloramphenicol, rifampin, tetracycline, trimethoprim/sulfamethoxazole, and vancomycin were determined by using the PASCO MIC gram-positive panel (Difco Laboratories, Detroit, MI), supplemented with horse blood. MICs for clindamycin, erythromycin, azithromycin, and clarithromycin were determined by using E-test strips (AB Biodisk, Solna, Sweden) on Mueller-Hinton agar supplemented with 5% horse blood incubated in an atmosphere containing 5% carbon dioxide. Phenotypes of macrolide resistance were differentiated according to the description of Seppala et al. (18) and Suttcliffe et al. (19). Resistance to both erythromycin and clindamycin indicated a constitutive type of resistance (CR), blunting of the clindamycin zone of inhibition proximal to erythromycin indicated an inducible type of resistance (IR), and susceptibility to clindamycin without blunting indicated the M-phenotype of resistance. For all antibiotics tested, the breakpoints suggested by the National Committee for Clinical Laboratory Standards were used (20,21).

At their physicians' discretion, eligible patients received a 10-day course of one of the following drugs: amoxicillin 75 mg/kg three times a day; amoxicillin/clavulanic acid 50 mg/kg twice a day; cefaclor 50 mg/kg twice a day; or clarithromycin 15 mg/kg twice a day. The attending physician was blinded to the results of microbiologic tests. Fisher's exact test and the chi-square test were performed by using Epi Info, version 6. For all tests, a p value of $\leq 0.05$ was considered statistically significant.

Six hundred children ages 1-13 years (median age 7.0) with acute pharyngitis were observed, and 180 (30%) whose throat cultures were positive for *S. pyogenes* were included in the study. Amoxicillin was prescribed to 42 patients, amoxicillin/clavulanic acid to 56, cefaclor to 35, and clarithromycin to 44. The clinical cure rates were 79.5% (35 of 44) in the clarithromycin group, 92% (39 of 42) in the amoxicillin group (p = 0.14 for comparison with clarithromycin), 100% (56 of 56) in the amoxicillin/clavulanic acid group (p = 0.0003 for comparison with clarithromycin), and 97.1% (34 of 35) in the cefaclor group (p = 0.03 for comparison with clarithromycin).

Results of post-treatment throat swabs were available from 159 patients. Bacterial eradication response rates were 77.2% (34 of 44) with clarithromycin, 88.8% (32 of 36) with amoxicillin group (p = 0.28 for comparison with clarithromycin), 95.8% (46 of 48) with amoxicillin/clavulanic acid (p = 0.03 for comparison with clarithromycin), and 90.3% (28 of 31) with cefaclor (p = 0.24 for comparison with clarithromycin). All 180 strains were susceptible to penicillin ($MIC_{90}$ <0.06 g/l) and other ß-lactams tested (Table 1). Overall, 69 (38.3%) of the 180 isolates were resistant to one or more macrolides, 7 (3.9%) to clindamycin, and 21 (11.6%) to the 16-member macrolide rokitamycin. Sixty-two percent (43 of 69 strains) of the erythromycin-resistant strains showed the M phenotype of resistance, 11.5% (8 strains) the CR phenotype, and 26.0% (18 strains) the IR phenotype.

Among the 159 patients, 19 (43.1%) of 44 treated with clarithromycin, 16 (44.4%) of 36 treated with amoxicillin, 13 (27.0%) of 48 treated with amoxicillin/clavulanic acid, and 8 (25.8%) of 31 treated with cefaclor had *S. pyogenes* resistant to erythromycin at the first swab collected before treatment.

*S. pyogenes* was eradicated in 12 (63.1%) of the 19 patients with erythromycin-resistant isolates and in 22 (88.0%) of 25 patients with erythromycin-susceptible isolates treated with clarithromycin (p = 0.07). As a control, the results of ß-lactam treatment were also studied. The rates of microbiologic eradication in patients with erythromycin-resistant isolates were 87.5% (14 of 16) for amoxicillin, 100% (13 of 13) for amoxicillin/clavulanic acid, and 100% (8 of 8) for cefaclor. Rates of microbiologic eradication for erythromycin-susceptible strains were 90% (18 of 20) for amoxicillin, 94.2% (33 of 35) for amoxicillin/clavulanic acid, and 86.9% (20 of 23) for cefaclor (p = 1.0; p = 1.0; p = 0.54, respectively, for comparison with erythromycin-resistant isolates).

In clarithromycin-treated patients, 6 of the 7 treatment failures were related to isolates with a CR phenotype (p = 0.0002 for comparison of percentages with other phenotypes of resistance, and p = 0.0001 for comparison with erythromycin-susceptible isolates) (Table 2).

Table 1. In vitro susceptibility[a] of *Streptococcus pyogenes* from 180 pharyngitis patients

| | | Erythromycin-resistant | | |
|---|---|---|---|---|
| | Erythromycin susceptible | M-phenotype | IR[b] | CR[c] |
| Erythromycin | 0.25 | 32 | 64 | >128 |
| Clarithromycin | 0.5 | 16 | 64 | >128 |
| Azithromycin | 0.25 | 16 | 64 | >128 |
| Clindamycin | 0.5 | 1 | 1 | >128 |
| Rifampin | 1 | 1 | 1 | 2 |
| Chloramphenicol | ≤4 | ≤4 | ≤4 | ≤4 |
| Ciprofloxacin | 0.25 | 1 | 2 | 2 |

[a]$MIC_{90}$ in g/ml; [b]IR = Inducible-type resistance; [c]CR = Constitutive-type resistance.

Table 2. Frequency of clarithromycin failure, by susceptibility profile

| | No. patients treated | No. treatment failed (%) | p value |
|---|---|---|---|
| Erythromycin-susceptible | 25 | 3 (12) | |
| Erythromycin-resistant | 19 | 7 (36.8) | 0.07[a] |
| CR[b] | 6 | 6 (100) | 0.0001[a] |
| other phenotypes | 13 | 1 (7.6) | 1.0[a]; 0.0002[c] |
| Total | 44 | 10 (22.7) | |

[a]For comparison of percentage of failure with erythromycin-susceptible; [b]CR = Constitutive-type resistance; [c]For comparison of percentage of failure between CR and other phenotypes of resistance.

### Conclusions

Our results show that in Genoa, 38% of *S. pyogenes* isolated from pharyngitis patients are erythromycin resistant. Sixty-three percent of such isolates belonged to a recently reported, noninducible M phenotype, described as having low-level resistance to erythromycin and sensitivity to clindamycin and 16-member macrolides (18). Twenty-six percent of resistant strains were classified as IR phenotype, characterized by low-level resistance to erythromycin and inducible resistance to 16-member macrolides and clindamycin after exposure to subinhibitory concentrations of erythromycin (5). The remaining isolates (11.5%) showed the CR phenotype, characterized by high-level resistance to macrolides and clindamycin.

Our study also examined whether in-vitro resistance could be a good predictor of clinical outcome in children with pharyngitis. Although physicians were instructed to choose the antibiotic without regard to clinical signs and symptoms, a bias due to selective antibiotic choice based on clinical presentation cannot be excluded. Clarithromycin was prescribed to 44 patients, 19 with erythromycin-resistant isolates and 25 with erythromycin-susceptible isolates. Although the rate of microbiologic eradication did not differ between patients with erythromycin-resistant isolates and those with erythromycin-susceptible isolates (63.1% vs. 88.0%; $p = 0.07$), a clear trend was observed toward a higher rate of eradication among erythromycin-susceptible isolates.

When results in clarithromycin-treated patients were analyzed by phenotype of resistance, the rate of treatment failure was 100% (6 patients) for CR phenotype, compared with 7.6% (1 of 13 patients) for other phenotypes ($p = 0.0002$) and 12% (3 of 25 patients) for erythromycin-susceptible isolates ($p = 0.0001$).

Failure of erythromycin to eradicate group A streptococci with high levels of resistance to erythromycin and lyncosamide has been reported (22,23). Seppala et al. (8), in a retrospective analysis of medical records, found that erythromycin failed in 47% of pharyngitis patients with erythromycin-resistant isolates, a rate significantly higher than the 4% observed in patients with erythromycin-susceptible isolates. The susceptibility profile of these strains, however, was consistent with phenotypes other than CR.

The eradication rate in patients with isolates belonging to phenotypes other than CR, thus showing low levels of resistance to macrolides, was comparable with that observed for erythromycin-susceptible isolates. However, our findings suggest that CR phenotype will be an accurate predictor of in-vivo failure of macrolides in the treatment of streptococcal pharyngitis. Whether the discrepancy between our results and those of a previous Finnish study (8) should be attributed to differing macrolides remains to be proven by large, well-controlled studies. Despite in-vitro cross-resistance with other 14-member macrolides, clarithromycin is characterized by elevated concentrations attained in different tissues (including tonsil tissue) because of its improved pharmacokinetic profile (5,24).

Because only a few alternative antimicrobial agents can be used to treat pharyngitis in patients allergic to ß-lactams, adequate interventions include a controlled use of macrolides and surveillance for the susceptibility of group A streptococci. Determining erythromycin resistance phenotypes seems to be a useful tool, particularly in areas where macrolides are frequently prescribed. Should the CR phenotype, reported infrequently at present, become prevalent, its high-level resistance may threaten the efficacy of macrolides and clindamycin in the treatment of streptococcal pharyngitis.

### Acknowledgments

The authors thank Antonio Di Biagio, Enrico Mantero, Sandra Ratto, and Maria Luisa Belli for their helpful comments and Roberto Ceccarelli for his review of the manuscript.

Dr. M. Bassetti is a physician in the Department of Infectious Diseases, University of Genoa, Italy. He works in Gaslini Children's Hospital, the largest children's hospital in Italy, and in San Martino teaching hospital. His research focuses on the clinical importance of antibacterial resistance and the application of cost-effective policies for antimicrobial use.

### References

1. Bisno AL. Group A streptococcal infections and acute rheumatic fever. N Engl J Med 1991; 325:783-93.
2. Kaplan EL. The resurgence of group A streptococcal infections and their sequelae. Eur J Clin Microbiol Infect Dis 1991;10:55-7.
3. Moses AE, Ziv A, Harari M, Rahav G, Shapiro M, Englehard D. Increased incidence and severity of *Streptococcus pyogenes* bacteremia in young children. Pediatr Infect Dis J 1995;14:767-70.
4. Peter G. Streptococcal pharyngitis: current therapy and criteria for evaluation of new agents. Clin Infect Dis 1992;14 Suppl 2:S218-23.

5. Schito GC, Pesce A, Marchese AJ. The role of macrolides in *Streptococcus pyogenes* pharyngitis. J Antimicrob Chemother 1997;39:562-5.
6. Lowbury EJL, Hurst L. The sensitivity of staphylococci and other wound bacteria to erythromycin, oleandomycin and spiramycin. J Clin Pathol 1959;12:163-4.
7. Maruyama S, Yoshiota H, Fujita K, Takimoto M, Satake Y. Sensitivity of group A streptococci to antibiotics. Am J Dis Child 1979;133:151-4.
8. Seppala H, Nissinen A, Jarvinen H, Huovinen S, Henriksson T, Herva E, et al. Resistance to erythromycin in group A streptococci. N Engl J Med 1992;326:292-7.
9. Hsueh PR, Chen HM, Huang AH, Wu JJ. Decreased activity of erythromycin against *Streptococcus pyogenes* in Taiwan. Antimicrob Agents Chemother 1995;39:2239-42.
10. Stingemore N, Francis GRJ, Toohey M, McGechie DB. The emergence of erythromycin resistance in *Streptococcus pyogenes* in Fremantle, Western Australia. Med J Aust 1989;150:626-31.
11. Gentry JL, Burns WW. Antibiotic resistant streptococci. Am J Dis Child 1980;133:801.
12. Garcia-Bermejo I, Cacho J, Orden B, Alos J, Gomez-Garces JL. Emergence of erythromycin-resistant, clindamycin-susceptible *Streptococcus pyogenes* isolates in Madrid, Spain. Antimicrob Agents Chemother 1998;42:989-90.
13. Orden B, Perez-Trallero E, Montes M, Martinez R. Erythromycin resistance of *Streptococcus pyogenes* in Madrid. Pediatr Infect Dis J 1998;17:470-3.
14. Cornaglia G, Ligozzi M, Mazzariol A, Valentini M, Orefici G, Fontana R. Rapid increase of resistance to erythromycin and clindamycin in *Streptococcus pyogenes* in Italy, 1993-1995. Emerg Infect Dis 1996;2:339-42.
15. Cocuzza C, Blandino G, Mattina R, Nicoletti F, Nicoletti G. Antibiotic susceptibility of group A streptococci in 2 Italian cities: Milano and Catania. Microb Drug Resist 1997;3:379-84.
16. Cornaglia G, Ligozzi M, Mazzariol A. Resistance of *Streptococcus pyogenes* to erythromycin and related antibiotics in Italy. Clin Infect Dis 1998;27 Suppl 1;S87-92.
17. Bassetti M, Mantero E, Gatti G, Di Biagio A, Bassetti D. *Streptococcus pyogenes* erythromycin resistance in Italy. Emerg Infect Dis 1999;5:302-3.
18. Seppala H, Nissinen A, Yu Q, Houvinen P. Three different phenotypes of erythromycin-resistant *Streptococcus pyogenes* in Finland. J Antimicrob Chemother 1993;32:885-91.
19. Sutcliffe J, Tait-Kamradt A, Wondrack L. *Streptococcus pneumoniae* and *Streptococcus pyogenes* resistant to macrolides but sensitive to clindamycin: a common resistance pattern mediated by an efflux system. Antimicrob Agents Chemother 1996;40:1817-24.
20. National Committee for Clinical Laboratory Standard Performance Standards for antimicrobial disk susceptibility tests. Document M2- A6. Villanova (PA): The Committee; 1997.
21. National Committee for Clinical Laboratory Method for dilution for antimicrobial susceptibility tests for bacteria that grow aerobically. Document M7- A4. Villanova (PA): The Committee; 1997.
22. Sanders E, Foster MT, Scott D. Group A beta-hemolytic streptococci resistant to erythromycin and lincomycin. N Engl J Med 1968;278:538-40.
23. Phillips G, Parratt D, Orange GV, Harper I, McEwan H, Young N. Erythromycin resistant *Streptococcus pyogenes*. J Antimicrob Chemother 1990;25:723-4.
24. Lode H, Boeckh M, Scaberg T. Human pharmacokinetics of macrolide antibiotics. In: Macrolides. Chemistry, pharmacology and clinical uses. Bryskier A, Butzler JP, Neu HC, Tulkens PM, editors. Paris: Arnette Blackwell; 1993; p. 409-20.
